# Differential presence of exons (DPE): sequencing liquid biopsy by NGS. A new method for clustering colorectal Cancer patients

**DOI:** 10.1186/s12885-022-10459-w

**Published:** 2023-01-03

**Authors:** David Rubio-Mangas, Mariano García-Arranz, Yaima Torres-Rodriguez, Miguel León-Arellano, Javier Suela, Damián García-Olmo

**Affiliations:** 1 Genómica y Medicina, NIMGenetics, Madrid, Spain. S. L, 28108 Madrid, Spain; 2 New Therapy Laboratory, Instituto de Investigación Sanitaria Fundación Jiménez Díaz, 28040 Madrid, Spain; 3 Department of Surgery, School of Medicine, Universidad Autónoma de Madrid, C. Arzobispo Morcillo, 4, 28029 Madrid, Spain; 4 Department of Surgery, Hospital Fundación Jiménez Díaz, 28040 Madrid, Spain; 5 Department of Surgery, New Therapies Laboratory, Foundation Health Research Institute-Fundación Jiménez Díaz University Hospital (FIIS-FJD), Universidad Autónoma de Madrid (UAM), Avda. Reyes Católicos, 2, 28040 Madrid, Spain

**Keywords:** Whole-exome sequencing, NGS, Cell-free DNA, DPE, Differential presence of exons, Colorectal cancer, Metastasis, Genometastasis

## Abstract

Differential presence of exons (DPE) by next generation sequencing (NGS) is a method of interpretation of whole exome sequencing. This method has been proposed to design a predictive and diagnostic algorithm with clinical value in plasma from patients bearing colorectal cancer (CRC). The aim of the present study was to determine a common exonic signature to discriminate between different clinical pictures, such as non-metastatic, metastatic and non-disease (healthy), using a sustainable and novel technology in liquid biopsy.

Through DPE analysis, we determined the differences in DNA exon levels circulating in plasma between patients bearing CRC vs. healthy, patients bearing CRC metastasis vs. non-metastatic and patients bearing CRC metastasis vs. healthy comparisons. We identified a set of 510 exons (469 up and 41 down) whose differential presence in plasma allowed us to group and classify between the three cohorts. Random forest classification (machine learning) was performed and an estimated out-of-bag (OOB) error rate of 35.9% was obtained and the predictive model had an accuracy of 75% with a confidence interval (CI) of 56.6–88.5.

In conclusion, the DPE analysis allowed us to discriminate between different patho-physiological status such as metastatic, non-metastatic and healthy donors. In addition, this analysis allowed us to obtain very significant values with respect to previous published results, since we increased the number of samples in our study. These results suggest that circulating DNA in patient’s plasma may be actively released by cells and may be involved in intercellular communication and, therefore, may play a pivotal role in malignant transformation (genometastasis).

**Supplementary Information:**

The online version contains supplementary material available at 10.1186/s12885-022-10459-w.

## Introduction

Colorectal cancer (CRC) is one of the most widespread malignancies and represents a challenge due to its high incidence and mortality worldwide [1]. Its burden is expected to increase by 60% with more than 2.2 million new cases and 1.1 million cancer deaths by 2030 [1]. Metastasis is the leading cause of death and prevention and early diagnosis are key to counteracting this trend [2].

The gold standard for the detection and diagnosis of CRC is colonoscopy. This approach allows for purely static analysis of the tumor at a given time and location, i.e. at the time of surgery [3]. However, CRC is a slowly progressive and dynamic disease, which becomes symptomatic when it progresses to advanced stages, with the timing of diagnosis being the most important factor influencing survival rates [4].

Fecal Occult Blood Test (FOBT) is the current preferred method for CRC screening in large populations [5]. Stool and blood tests to identify methylated DNA are rising as the preferred genetic-based methods for screening pre-symptomatic CRC patients [6], but have important limitations such as cost, standardization, and high false negative and positive results [7]. Therefore, there is a need to develop new early detection methods and to develop non-invasive methods to detect CRC at earlier stages, as well as to improve and incorporate new detection methods at more advanced stages in order to introduce Precision Medicine Criteria in the follow-up of these patients. The search for specific markers appears to be essential in improving the management of CRC patients along disease phases: from diagnosis to treatment and follow-up.

Liquid biopsy, by means of tumoral cell-free DNA (cfDNA) detection, has been one of the most encouraging expectations of cancer monitoring during the last two decades, providing additional information and enabling the discovery of new biomarkers [8]. In recent years, circulating tumoral cfDNA has proven to be the most appropriate non-invasive method and the best way to analyze tumors specially when a biopsy to obtain tumor tissue was difficult or not available [9]. Most studies suggest that cfDNA analysis should be used for molecular profiling, therapy-related mutation detection and minimal residual disease [10–12]. Moreover, a recent study showed how a circulating tumor DNA (ctDNA) -guided approach to the treatment of stage II colon cancer reduced adjuvant chemotherapy use without compromising recurrence-free survival [13].

Circulating tumor DNA (ctDNA) is a plasma biomarker widely used in oncology [14, 15]. ctDNA detection in colorectal cancer is related to RAS/BRAF point mutations before anti-EGFR treatment [16–18]. Moreover, increased cfDNA level, together with a heterogeneous hotspot mutation pattern, provide a strong clinical prognostic predictor.

Although droplet digital PCR (ddPCR) seems to be the most used technology to analyze cancer-specific mutations [19], the advent of next-generation sequencing (NGS) and new bioinformatic methods make it possible to use more complex liquid biopsy analysis, leading to what is known as precision medicine. Our group has gone further, developing an approach based on whole exome sequencing in plasma called “differential presence of exons” (DPE).

DPE is a new and innovative strategy of NGS analysis with the evaluation of differential presence of exons in cfDNA to cluster and classifies patients with disseminated and localized disease [20]. The DPE method for clustering showed to be easier and more cost effective than other NGS methods with the same task [20]. This new approach was alto tested in an animal model of CRC showing similar results [21].

The target of the present study is to expand DPE analysis by NGS comparing healthy to patients bearing CRC at different stages, in order to explore the genomic heterogeneity of cfDNA and to search for exonic signatures that can be used in precision medicine.

## Materials and methods

### Patients selection

A ninety-six CRC patients cohort was recruited from April 2018 to November 2019 in the Department of General Surgery at the University Hospital Fundación Jiménez Díaz, Madrid, Spain. All patients underwent proper informed consent and the study received approval by the hospital clinical research ethical committee (Cod ER_PIC_135/2017_FJD). Inclusion and exclusion criteria are shown in Table 1.

**Table 1:** Criteria for patient selection

	Non-metastatic cohort (N)	Metastatic cohort (M)	Unclassifiable cohort (U)
**Inclusion criteria**	Age > 18 years		
	Candidates for elective surgery		
	Provided informed consent		
	Histological diagnosis: colon adenocarcinoma of enteroid pattern		
	• pT1-pT3	• Any T or N	• pT4 and/or pN1-pN2
	• pN0	• M1: liver metastasis with an enteroid adenocarcinoma pattern, established histologically	• M0 (established by PET-CT)
	• M0 (established by PET-CT)		
	• R0		
**Exclusion criteria**	Previous cancers in other locations		
	Lynch syndrome or other hereditary intestinal cancers		

Patients were distributed into three groups: nonmetastatic colon cancer (N; *n* = 68), metastatic colon cancer (M; *n* = 17) and unclassified patients according to the selection criteria (U; *n* = 11). Patient’s clinical characteristics are shown in Table 2.

**Table 2:** Clinical-biological characteristics

Age (mean years)	70 (range 23–101)	N (%)
**Sex**	Males	53 (55%)
	Females	43 (45%)
**Histologic grade of the tumor**	Low grade (Well-differentiated tumors and moderately differentiated tumors)	86 (89.59%)
	High grade (Poorly differentiated tumors)	3 (3.12%)
	N/A	7 (7.29%)
**Stage at diagnosis**	I	7 (7.29%)
	II	20 (20.83%)
	III	45 (46.88%)
	IV	21 (21.88%)
	N/A	3 (3.12%)
**Tumour location**	Sigmoid	43 (44.79%)
	Right	25 (26.04%)
	Left	7 (7.29%)
	Transverse	4 (4.17%)
	Rectum	10 (10.42%)
	Splenic	2 (2.08%)
	N/A	5 (5.21%)
**Follow-up**		6 years ±7 months
**Molecular Data**	*RAS* mut	6 (6.25%)
	Not *RAS* mut	4 (4.17%)
	N/A *Ras mut*	86 (89.58%)
	*BRAF*^V600E^	3 (3.12%)
	Not *BRAF*^V600E^	8 (8.33%)
	N/A *BRAF*^V600E^	85 (88.54%)
	Microsatellite instability (MSI)	7 (7.29%)
	Not Microsatellite instability (MSI)	60 (62.50%)
	N/A MSI	29 (30.21%)

N = number of patients. N/A = not applicable or not available

On the other hand, 63 volunteer healthy donors (H) were enrolled in the study by providing informed consent and approval by the hospital clinical research ethical committee. Blood samples were obtained through vein puncture from the biobank of the University Hospital Fundación Jiménez Díaz, Madrid, Spain.

### cfDNA extraction from plasma samples

Ten millilitres of peripheral blood were collected from each patient before surgery at room temperature into a Streck cell-free circulating DNA (cfDNA) BCT® (Streck, La Vista, NE, US) tubes and processed immediately (less than 1 hour). Samples were centrifuged at 1800×g for 10 minutes and the plasma obtained in the first centrifugation was centrifuged again at 3000×g at 4 °C for 10 minutes [20], aliquoted and stored at − 80 °C prior to analysis. Plasma cDNA was extracted automatically using a modified protocol of the QiaSymphony DSP circulating DNA kit on the QIAsymphony (QIAGEN, Hilden, Germany). cfDNA was quantified using the Qubit dsDNA HS assay kit (Thermo Fisher Scientific Inc., Waltham, MA, USA) and stored at − 20 °C.

### Whole exome sequencing

An optimized WES protocol for cfDNA samples was performed using the Twist Human Core Exome kit + RefSeq V1 (Twist Bioscience Corporation, San Francisco, CA, USA), focused on clinically relevant genes with 41.2 Mb capture size.

Samples were barcoded (unique dual index), qualified and quantified using TapeStation 2200 (Agilent Technologies, Santa Clara, CA, USA) and Qubit 2.0 Fluorometer (ThermoFisher Scientific, Waltham, MA, USA).

Illumina NovaSeq 6000 system (Illumina, Inc., San Diego, CA, USA) was used for 100 bp pair-end read sequencing. Reads were aligned against GRCh38.103 human genome build using Bowtie-2 aligner [22].

### Data analysis

#### Detection of differentially present exons (DPE)

DPE analysis was done with ‘EdgeR’ R package [23], using the reads counts by exon that were calculated with HTSeq-count [24]. Sequence data was normalized and filtered for exons with less than 1 count per million (CPM) in at least 20 samples. Edger’s background statistical methods were based on generalized linear models (glm), which test for DPE using either likelihood ratio tests (LRT) [23] or quasi-likelihood F-tests (QLF) [25]. Exons with a False Discovery Rate (FDR) ≤ 0.001 were selected for each method. Finally, common exons highlighted by both methods were considered as DPE. A Venn diagram was made to detect the exons in common for the different comparisons of the study. Venny 2.1 (https://bioinfogp.cnb.csic.es/tools/venny/index.html) virtual tool was used for the Venn diagrams.

#### DPE: clustering and principal components analysis (PCA)

Three comparisons were performed to obtain a DPE signature for each of the comparisons:

1. Patients with metastatic colorectal cancer (M) vs patients with non-metastatic colorectal cancer (N).
2. Cancer patients (C) vs. healthy controls (H).
3. Patients with metastatic colorectal cancer (M) vs. healthy controls (H).

PCA was performed using the DPE resulting from the above filters and using an in-house script. A Venn diagram was performed for the three comparisons in order to obtain a common signature that could explain the differences in DPE. After obtaining the exons in common for the three comparisons-, a clustering pooled using Ward’s method [26] and principal component analysis was performed for the three comparisons to see how these exons behaved.

#### Random forest

Random forest (RF) classification was implemented with an R script using the “randomForest” package [27]. To generate a predictive model, 16 metastatic samples, 67 non-metastatic samples and 62 healthy controls were selected as training sets, because the outlier healthy control and two patients with NA values in some of the 510 exons were previously eliminated. The mean value of the probabilities obtained was calculated. The accuracy of the resulting model was tested by checking its ability to correctly classify the randomly drawn samples into their corresponding groups of origin. In addition, the 11 unclassifiable samples were tested to see in which group they were classified.

On the other hand, we used the dataset to extract random samples for training and for test (70 and 30% respectively) using 5000 trees and using all the variables except those with some NA value that were previously eliminated in the random forest process.

#### Gene list functional enrichment analysis - pathway analysis

Ensemble biomart platform (https://www.ensembl.org/info/data/biomart/index.html) was used to highlight a gene list from DPE [28]. Functional analysis in gene list was performed Enrichr (https://maayanlab.cloud/Enrichr/#) [29], Genecodis 4.0 (https://genecodis.genyo.es/) [30] and ShinyGO V0.65 (http://bioinformatics.sdstate.edu/go/) [31].

Enrichr was used to analyze gene ontology (GO) [32] and KEGG [33].

### Statistical analysis

Nonparametric Kolmogorov–Smirnov and Kruskal-Wallis test for significance were performed in R to test differences in DNA concentration in plasma, histologic grade and TNM stratification (cutoff *P*-value of *P* < 0.05). LRT and QLF tests were performed for DPE with a FDR of ≤0.001.

### Data availability

Raw whole exome sequencing data of the samples in the study were deposited at European Genome-phenome Archive (EGA) under accession number EGAP00001002916 (https://wwwdev.ebi.ac.uk/ega/studies/EGAS00001006656).

## Results

### Data analysis

On average, 129 million paired-end reads per sample were collected after sequencing, with a minimum of 76 million and a maximum of 241 million reads. Read depth varied in a range from 105x to 333x, with an average read depth of 196x per sample.

### Exploratory data analysis. Clinical data

After performing the corresponding filters, the results were visualized in a multidimensional scaling plot (MDSplot) and the samples were separated according to the sex of the patient. (Supplementary Fig. S1). To avoid this bias, an internal script was developed to remove those sexual exons from the analysis and an MDSplot was re-run for each of the available clinical situations (Supplementary Fig. S2). We observed that the samples were not distributed according to TNM stratification, disease stage, histologic grade, presence of MSI, BRAF and RAS mutations and age.

The age of the patients and the results for each study group (metastatic, non-metastatic, unclassified) are shown in supplementary Table S1.

### DPE analysis. Clustering and principal component analysis

The differential presence of exons was analyzed with edgeR, using the QLF and LRT tests, with a threshold of FDR ≤ 0.001. The following comparisons were performed: metastatic vs non-metastatic, cancer vs healthy and metastatic vs healthy.

#### Metastatic vs non-metastatic patients

**Fig. 1:**
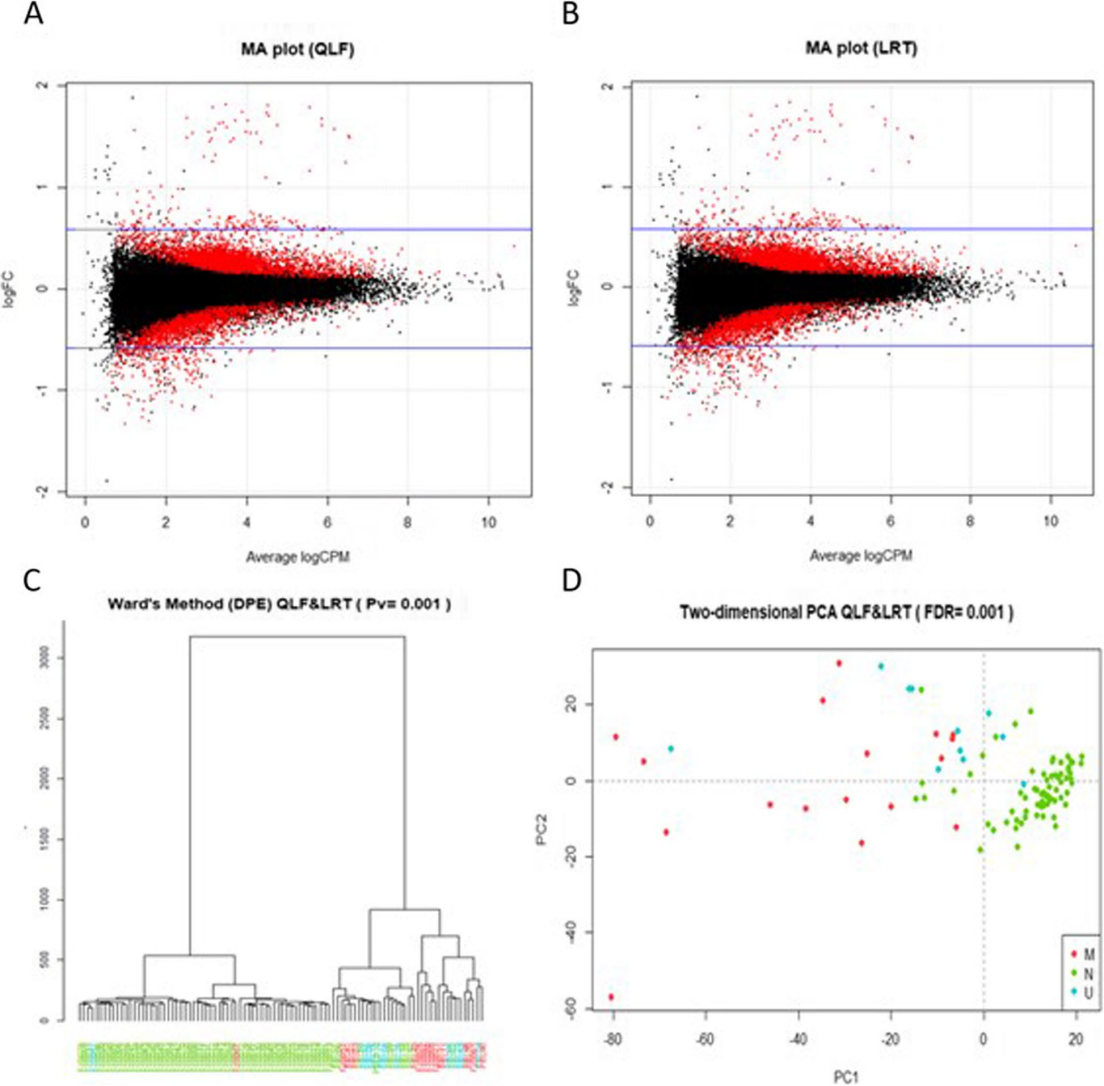
Exploratory analysis of patients using DPE (M vs N). MA plots for selected differentially present exons combining two different methods: quasi-likelihood F-tests (QLF) (**A** plot) and likelihood ratio tests (LRT) (**B** plot) for comparative M vs N. The log-fold change (FC) ratio is plotted on the y-axis, and the average normalized counts (counts per million; CPM) is plotted on the x-axis. Differentially present exons are highlighted in red (DPE; *p* ≤ 0.001) and a total of 1760 exons were obtained with EdgeR. In the graph (**C**) we can see grouping of patients using normalized values of differentially present exons (DPE) by Ward’s method. Patients are marked with different colors according to the group in which they were included: M: red; N: green; and U: blue. Most metastatic (M) and non-metastatic (N) patients were clearly separated into two groups, while unclassifiable (U) patients were located between M and N, indicating that they share features with both groups. In the two-dimensional (**D**) plot, a principal component analysis (PCA) can be observed. Metastatic (M) and non-metastatic (N) patients cluster in a cloud of different points and are separated from each other, while unclassifiable (U) patients are located between M and N, probably because of their intermediate characteristics. M: red; N: green; and U: blue

For the metastatic (M) vs non-metastatic (N) group comparison, a total of 1760 differentially present exons were obtained, common between the QLF and LRT method with FDR ≤ 0.001, of which 1405 overrepresented in the M group and 355 overrepresented in the N group. The MA plots for selected DPEs are shown in Fig. 1A-B. Statistically significant exons were located at the margins of the point cloud, as expected.

Normalized DPEs clustering was performed using Ward’s method, which gives a distance tree shown in Fig. 1C with the 1760 DPEs. For clustering, unclassifiable samples were also included to see how they were distributed in the tree. Samples were mainly clustered into disease groups (metastatic, non-metastatic and unclassified). PCA was then performed as can be seen in Fig. 1D, which shows a two-dimensional plot with the first two principal components. As can be seen, the M and N groups are clearly separated and correctly grouped; unclassifiable samples were also included to see their distribution in the graph.

#### Cancer patients vs healthy donor analysis

For the cancer patients vs. healthy donor comparison, a total of 14,300 DPE were obtained, common between the QLF and LRT method with FDR ≤ 0.001. Despite this, the fold changes (lgFC) were much lower than in the M vs N comparison, thus indicating that the changes found were small.

**Fig. 2:**
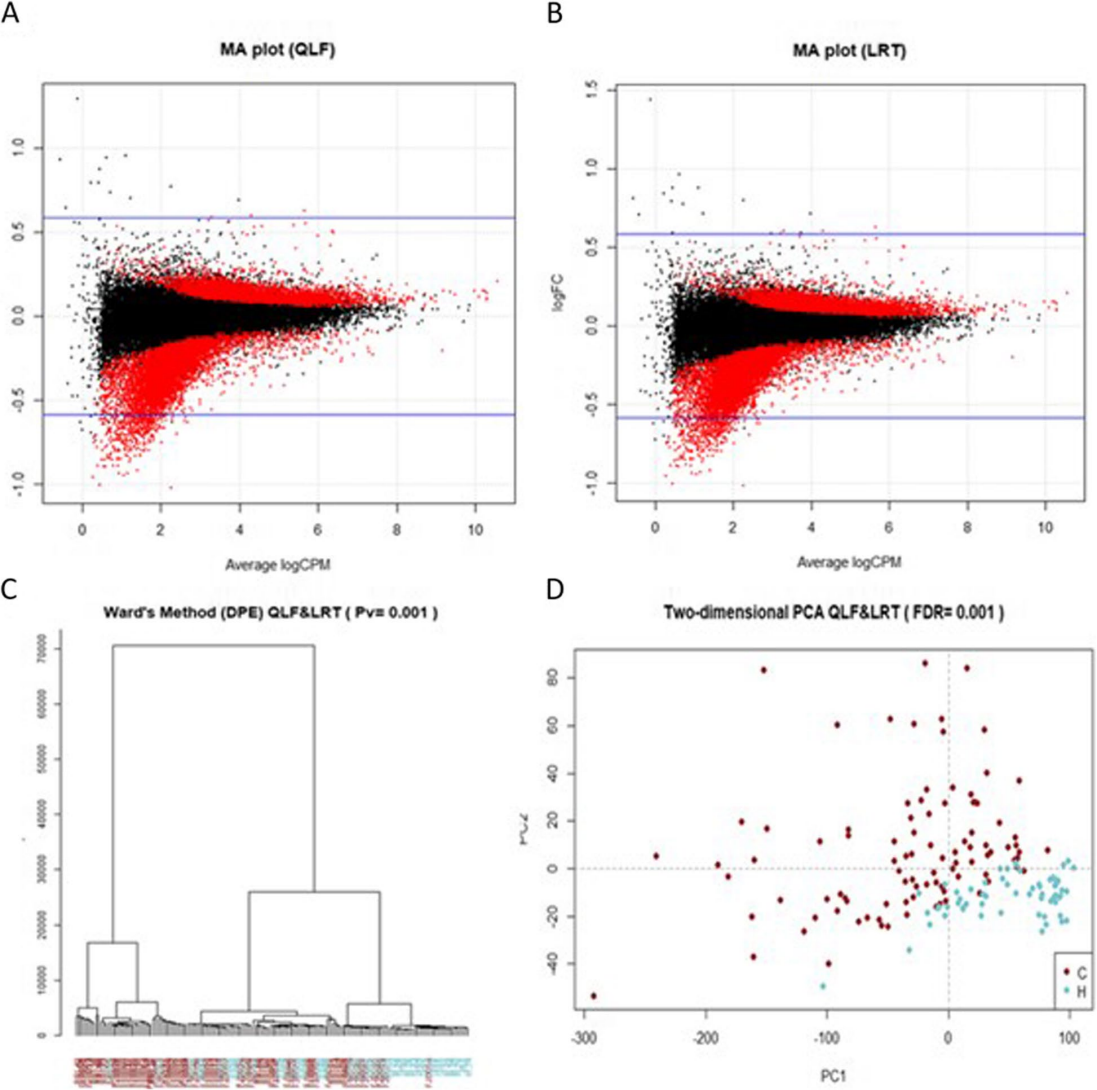
Exploratory analysis of patients using DPE (C vs H). MA plots for selected differentially present exons combining two different methods: quasi-likelihood F-tests (QLF) (**A** plot) and likelihood ratio tests (LRT) (**B** plot) for comparative C vs H. The log-fold change (FC) ratio is plotted on the y-axis, and the average normalized counts (counts per million; CPM) is plotted on the x-axis. Differentially present exons are highlighted in red (DPE; *p* ≤ 0.001) and a total of 14,300 exons were obtained with EdgeR. In the graph (**C**) we can see the grouping of patients using the normalized values of differentially present exons (DPE) by Ward’s method. Patients are marked with different colors according to the group in which they were included: C: brown and H: blue. Some of the cancer patients were clearly separated from the healthy ones. However, some of the cancer patients and healthy controls appear grouped together. A principal component analysis (PCA) can be seen in the two-dimensional (**D**) plot. Cancer patients (C) and healthy controls (H) where separated in a cloud of individual points. However, some patients and healthy controls appear together in the same point cloud, corresponding to non-metastatic patients who have a small tumor. C: cancer - brown and H: healthy - blue

Of the 14,300 DPE’s of which 5663 were overrepresented in the cancer group and 8637 in the healthy group, MA plots for the selected DPE’s as shown in Fig. 2A-B. Statistically significant exons were located at the margins of the point cloud, as expected.

As in the previous case, the clustering of the normalized DPEs were performed using Ward’s method, which gave a distance tree shown in Fig. 2C with the 14,300 DPEs. The samples clustered sparsely, although it was seen that most of the cancer patients clustered together such as most of the healthy controls, although some of the N cancer samples tended to cluster together with the healthy ones. We then performed PCA with the DPEs in common (QLF and LRT test) with an FDR less than 0.001. Fig. 2D shows a two-dimensional plot with the first two principal components, showing two-point clouds, one for cancer patients and one for healthy donors, although some samples from the two groups in the study clustered together.

#### Metastatic cancer patients vs healthy donor analysis

**Fig. 3:**
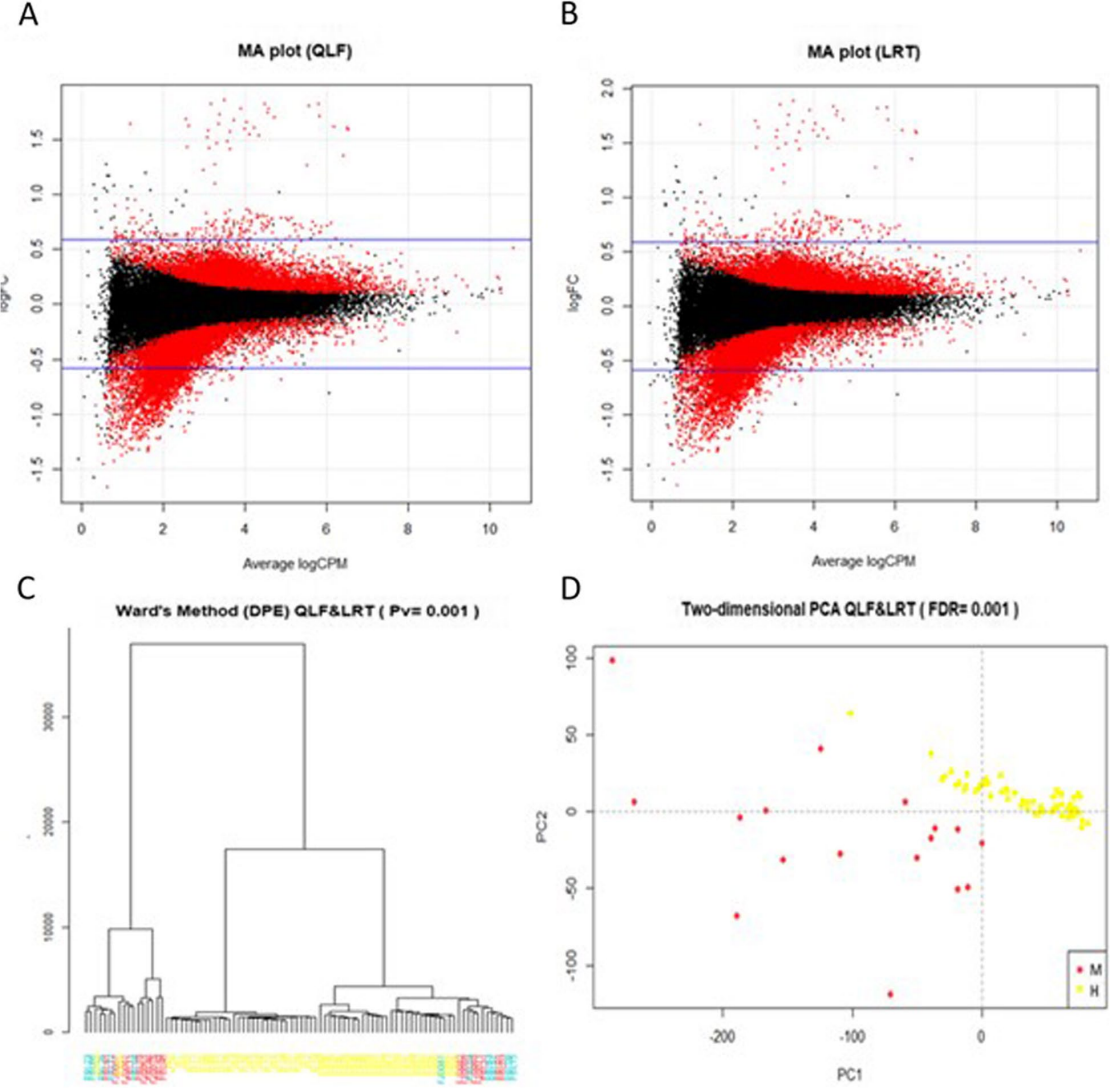
Exploratory analysis of patients using DPE (M vs H). MA plots for selected differentially present exons combining two different methods: quasi-likelihood F-tests (QLF) (**A** plot) and likelihood ratio tests (LRT) (**B** plot) for comparative M vs H. The log-fold change (FC) ratio is plotted on the y-axis, and the average normalized counts (counts per million; CPM) is plotted on the x-axis. Differentially present exons are highlighted in red (DPE; *p* ≤ 0.001) and a total of 14,430 exons were obtained with EdgeR. In the graph (**C**) we can see the grouping of the participants using the normalized values of differentially present exons (DPE) by Ward’s method. The participants are marked with different colors according to the group in which they were included: M: red, U: blue and H: yellow. Some of the metastatic patients were clearly separated from the healthy ones. However, the metastatic patients appear at the ends of the tree and the healthy controls in the middle, and the unclassifiable near the metastatic patients. A principal component analysis (PCA) can be seen in the two-dimensional (**D**) plot. Metastatic patients (M) and healthy controls (H) appear separated in a cloud of individual points. M (red) and H (yellow)

In the comparison of metastatic colorectal cancer patients vs. healthy controls, a total of 14,430 DPE’s were obtained, common between the QLF and LRT method with FDR ≤ 0.001, of which 6526 overrepresented in the M group and 7904 overrepresented in the H group. The MA plots for selected DPEs are shown in Fig. 3A-B. Statistically significant exons were located at the margins of the point cloud, as expected.

Clustering of the normalized DPEs was performed, as in the previous cases, using Ward’s method, which gave a distance tree shown in Fig. 3C with the 14,430 DPEs. For clustering, unclassifiable samples were also included to see how they were distributed in the tree. The samples were grouped at the extremes mainly those M patients and some indeterminate and a few healthy controls. Healthy controls were mainly grouped together in the middle. PCA was then performed, as can be seen in Fig. 3D, which showed a two-dimensional plot with the first two principal components. M and healthy groups were clearly separated and correctly grouped.

#### Integration of the three groups (M, N, H)

**Fig. 4:**
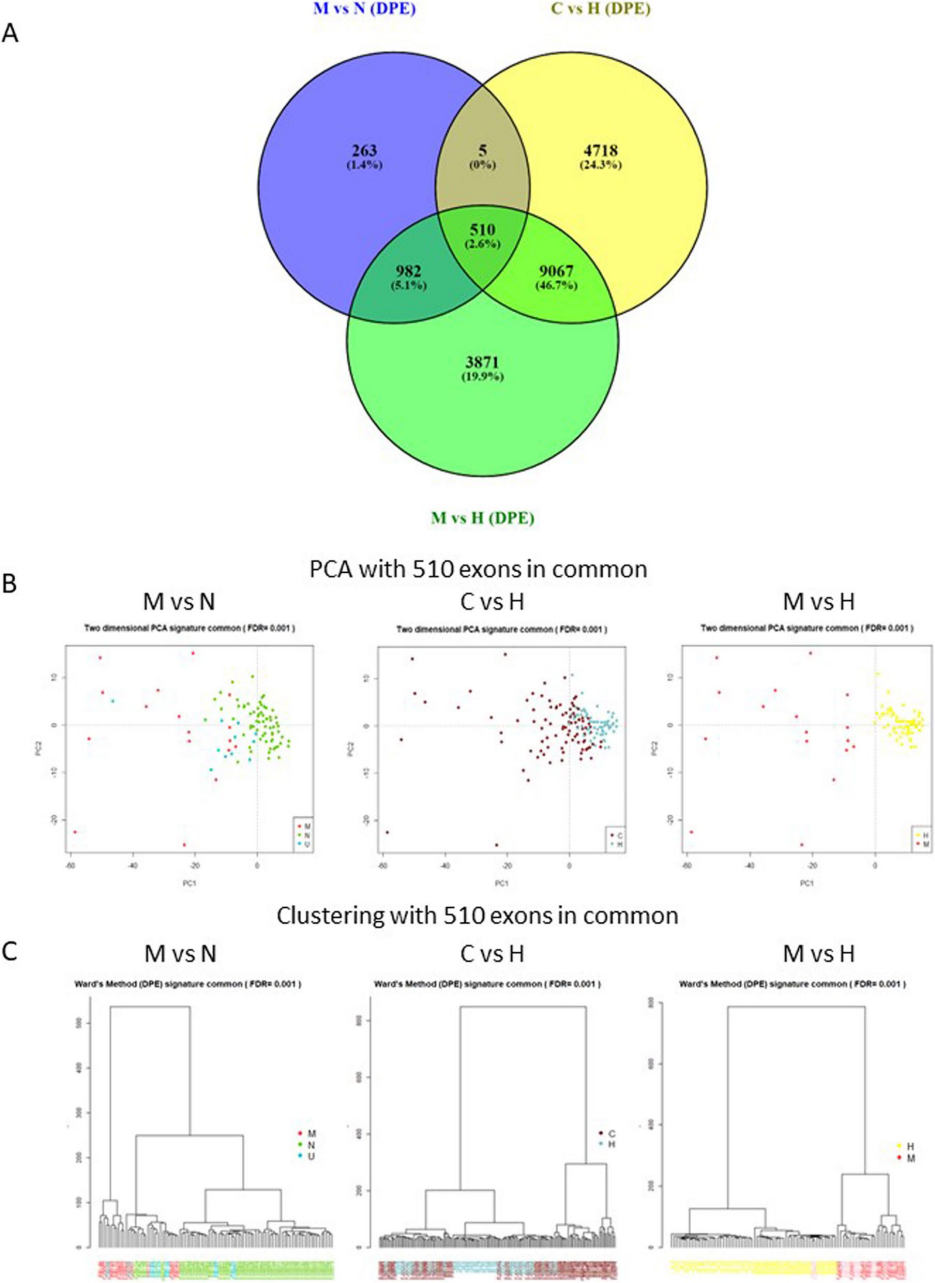
Exploratory analysis of common exons in the three groups of patients studied. A Venn diagram of all comparisons using the Venny platform; Oliveros, J.C. (2007–2015) Venny. An interactive tool for comparing lists with Venn’s diagrams. https://bioinfogp.cnb.csic.es/tools/venny/index.html. As we can observe, there are 510 exons in common from the three comparatives (M vs N, C vs H and M vs H) and these exons were used an exonic signature related to colorectal cancer. **B** Two-dimensional principal component analysis (PCA) plot with the 510 exons in common (exonic signature). Using samples from the comparative M vs N, C vs H and M vs H. It can be seen that there is discrimination between the groups using these 510 exons. Samples from groups M, N, U and H are marked with different colors according to the graph. The graph on the left: M in red, N in green, and U in blue. The middle graph: C in brown and H in blue. The graph on the right: M in red and H in yellow. **C** Clustering of the samples by Ward’s method with the 510 exons in common (exonic signature). We can see the clustering on the left: M in red, U in blue and N in green. It is clear that there is separation between the clusters. In the middle cluster: C in brown and H in blue, the separation between groups is also observed and in the cluster on the right: M in red, U in pink and H in yellow, the separation between groups is also observed. Unclassifiable patients (U) were placed in the center, indicating that they share features with both groups. M; Metastatic, N; Non-metastatic, C: Colorectal cancer patients and H; healthy controls

After performing an integration of the three comparatives of the study using a Venn diagram (Fig. 4A) and the QLF/LRT tests, we obtained a signature of 510 exons that could have biological value. This list was used to perform a supervised separation analysis between M, N and healthy groups (Fig. 4B-C).

We succeeded to segregate M patients from healthy controls, although the separation between cancer and healthy was not entirely possible. Most of the N patients were clustered together, while the M patients were more scattered.

We then examined in which genes were located those exons. Considering that some exons belonged to the same gene, we finally identified a total of 382 genes. We then also used these exons in common among the three comparisons to perform pathway enrichment analysis.

### Random forest

These results encouraged us to develop a predictive algorithm to classify the samples using an internal random forest script. To do so, we performed two approaches:

1) Classification of unclassifiable samples, the results of which are shown in supplementary Table S2.

2) Divide the dataset into two study populations (training 70%) and (testing 30%) and perform a random forest with a ntree of 5000 and a mtry of 510, obtaining a 35.9% OBB error rate and a 75% accuracy. The sensitivity and classification accuracy of all MCC, NMCC and healthy groups metastatic for the test dataset is shown in Supplementary Table S3.

### Pathway analysis

**Fig. 5:**
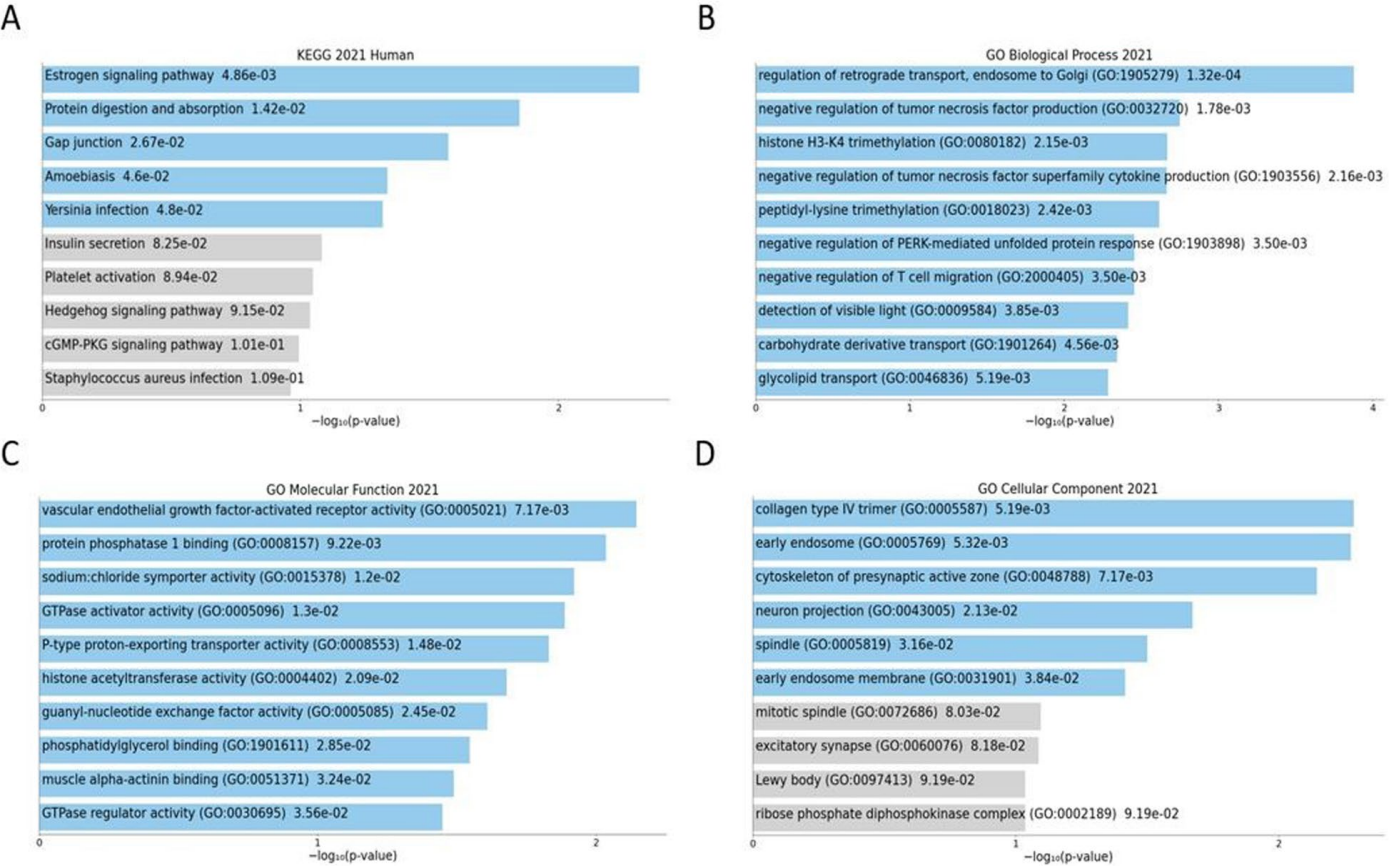
Pathways analysis. **A** Bar chart of top enriched terms from the KEGG_2021_Human gene set library. The top 10 enriched terms for the input gene set are displayed based on the -log10(p-value), with the actual *p*-value shown next to each term. The term at the top has the most significant overlap with the input query gene set. **B** Bar chart of top enriched terms from the GO_Biological_Process_2021 gene set library. The top 10 enriched terms for the input gene set are displayed based on the -log10(p-value), with the actual p-value shown next to each term. The term at the top has the most significant overlap with the input query gene set. **C** Bar chart of top enriched terms from the GO_Molecular_Function_2021 gene set library. The top 10 enriched terms for the input gene set are displayed based on the -log10(*p*-value), with the actual *p*-value shown next to each term. The term at the top has the most significant overlap with the input query gene set. **D** Bar chart of top enriched terms from the GO_Cellular_Component_2021 gene set library. The top 10 enriched terms for the input gene set are displayed based on the -log10(*p*-value), with the actual *p*-value shown next to each term. The term at the top has the most significant overlap with the input query gene set

The pathway study (KEGG and Gene Ontology - GO) at that signature revealed an enrichment of pathways that could be associated with cancer with the Enrichr tool. However, we did not obtain significant results for the 510 exons when we corrected the *p*-value by multiple testing (adjusted *p*-value). The genes were related to three biological functions according to the KEGG 2021 Human database; Estrogen signalling pathway (*p*-value = 0,004), protein digestion and absorption (*p*-value = 0,01) and Gap junction (*p*-value = 0,02) (Fig. 5A).

The most overrepresented gene ontology pathways were as follows:

**-** Biological Processes (BP): regulation of retrograde transport, endosome to Golgi (GO:1905279) (*p*-value = 1.3E-4), negative regulation of tumor necrosis factor production (GO:0032720) (*p*-value = 0.001) and histone H3-K4 trimethylation (GO:0080182) (*p*-value = 0.002). It is worth mentioning the fourth most significant pathway known as negative regulation of tumor necrosis factor superfamily cytokine production (GO:1903556) (*p*-value = 0,002), which is related to cancer (Fig. 5B).

**-** Molecular Functions (MF): vascular endothelial growth factor-activated receptor activity (GO:0005021) (*p*-value = 0,007), protein phosphatase 1 binding (GO:0008157) (*p*-value = 0,009) and sodium:chloride symporter activity (GO:0015378) (*p*-value = 0,01) (Fig. 5C).

- Cellular Components (CC**)**: collagen type IV trimer (GO:0005587) (*p*-value = 0,005), early endosome (GO:0005769) (*p*-value = 0,005) and cytoskeleton of presynaptic active zone (GO:0048788) (*p*-value = 0,007) (Fig. 5D).

We performed a functional analysis with the ShinyGO V0.65 tool of the 382 genes with all the Pathway database incorporated in the tool and obtained significant results (FDR < 0.005) with pathways related to cancer Supplementary Table S4. We also observed that our gene list was enriched in genes found on chromosome 20 as can be seen in Supplementary Fig. S3.

Finally, the genecodys platform was used to perform the functional analysis with the panther pathways, bioplanet and wikipathways databases (Supplementary Fig. S4):

**-** Panther pathways, the three most significant pathways were: 5HT3 type receptor mediated signaling pathway (*p*-value = 0,03), angiotensin II-stimulated signaling through G proteins and beta-arrestin (*p*-value 0,02) and integrin signaling pathway (*p*-value = 0,02).

**-** Bioplanet, the three most significant pathways were: collagen biosynthesis and modifying enzymes (*p*-value = 0,002), Ion transport by P-type ATPases (0,002) and Extracellular matrix organization (*p*-value = 0,003).

**-** Wiki pathways, the three most significant pathways were: circadian rhythm genes (*p*-value = 0,001), hippo signaling regulation pathways (*p*-value = 0,004) and G protein signaling pathways (*p*-value = 0,004).

## Discussion

Next-generation sequencing (NGS) has been proposed as a suitable tool for liquid biopsy in colorectal cancer (CRC), although most studies to date have focused on sequencing panels of potential candidate genes that are clinically actionable [34] . Mutation analysis by liquid biopsy are affected by several issues, including, among others: cfDNA concentration [35], ctDNA abundance in plasma (related to tumor mass), and/or tumor heterogeneity [36]. To solve these issues that affect sensitivity, high sequencing depths are needed, being a costly strategy in expanded NGS panels.

In this study,, we have tested a new approach called differential presence of exons (DPE), previously described by the Garcia-Olmo research group at the University Hospital Fundación Jiménez Díaz [20, 21]. This NGS approach at a relatively shallow depth represents an easy, rapid, non-invasive and affordable strategy for future use in clinical practice.

In this DPE scenario, we developed a more novel and robust technology to draw a signature in common between metastatic and non-metastatic CRC patients and healthy controls. We were able to define differential DPE signatures between the three groups (metastatic, non-metastatic, healthy) consisting of 510 exons that could explain the overall variation.

The resulting common DPE profile was used to cluster and classify all study participants and this information was processed to develop a DPE algorithm generating a predictive model using machine learning. Most patients were correctly grouped and separated between metastatic and non-metastatic, and it was also observed that when using these 510 DPEs healthy controls were placed in a point cloud in the PCA. Unclassifiable patients were clustered between non-metastatic and metastatic groups according to the common features they share.

Differential detection of exons suggests differential release of cfDNA actively by tumor and non-tumor cells, which could have biological implications by acting as a means of intercellular communication. Previous studies have described that cell-free nucleic acids circulating in the plasma of patients with colorectal cancer induce oncogenic transformation of distant cells, predisposing them to malignant transformation (genometastasis theory) [37, 38]. Moreover, It has been suggested that metastatic seeding may occur before clinical detection [39] and cfDNA may be involved in the metastatic process [40]. This leads us to believe that this DPE signature may have some involvement in malignant transformation and metastasis.

In fact, functional analysis of the 510 DPEs highlighted significant results in cancer related pathways such as kidney cancer [41], liver cancer [42] and estrogen signaling (all of them related to oncogenic development and associated with CRC) [43, 44]. Examples of CRC related Gene Ontology also associated with colorectal cancer were GO:0005021 (vascular endothelial growth factor receptor activity) [45] and GO:0032720 (negative regulation of tumor necrosis factor production) [46]. Therefore, DPE discovery not only may contribute to elucidate the molecular mechanism of carcinogenesis, but also provides a new approach to liquid biopsy analysis and proposes the use of DPE as a non-invasive biomarker.

To date, this is the largest dataset using DPE for CRC, we have included healthy controls that were not included in previous studies. We have also innovated in the use of new exome technology, as well as implemented a new standardised cfDNA extraction protocol that could facilitate its incorporation into clinical practice.

To conclude, it should be commented that liquid biopsy analysis can be used to gain new insights into the biology of metastasis and as a companion diagnostic to improve the stratification of therapies and to obtain information on therapy-induced cancer cell selection (precision medicine) and the technical and clinical validation of assays is very important [47].

## Conclusions

The DPE-based approach has allowed us to discriminate between patients with cancer and healthy controls, patients with metastatic CRC and healthy controls, and a tendency in patients with metastatic and non-metastatic CRC. From all comparisons we have obtained a common signature of 510 exons and corroborates with previous results of discrimination between groups. Functional analysis of the resulting signature (510 DPEs) is associated with cancer-associated pathways. The results found here support the theory of an active and targeted release of cfDNA (genometastasis). For future studies, we need more samples to profile this signature and predict the prognostic value of survival of these patients.

## Supplementary Information

**Additional file 1.**

**Additional file 2.**

**Additional file 3.**

**Additional file 4.**

**Additional file 5.**

## Data Availability

The datasets supporting the conclusions of this article are available in the European Genome Archive (EGA) repository, under data accession number EGAP00001002916 (https://wwwdev.ebi.ac.uk/ega/studies/EGAS00001006656).
