# Supplementary figures and images for: Differential presence of exons (DPE): sequencing liquid biopsy by NGS. A new method for clustering colorectal Cancer patients
Source: BMC Cancer. 2023 Jan 3;23:2. doi: 10.1186/s12885-022-10459-w (PMC9808981; doi:10.1186/s12885-022-10459-w)

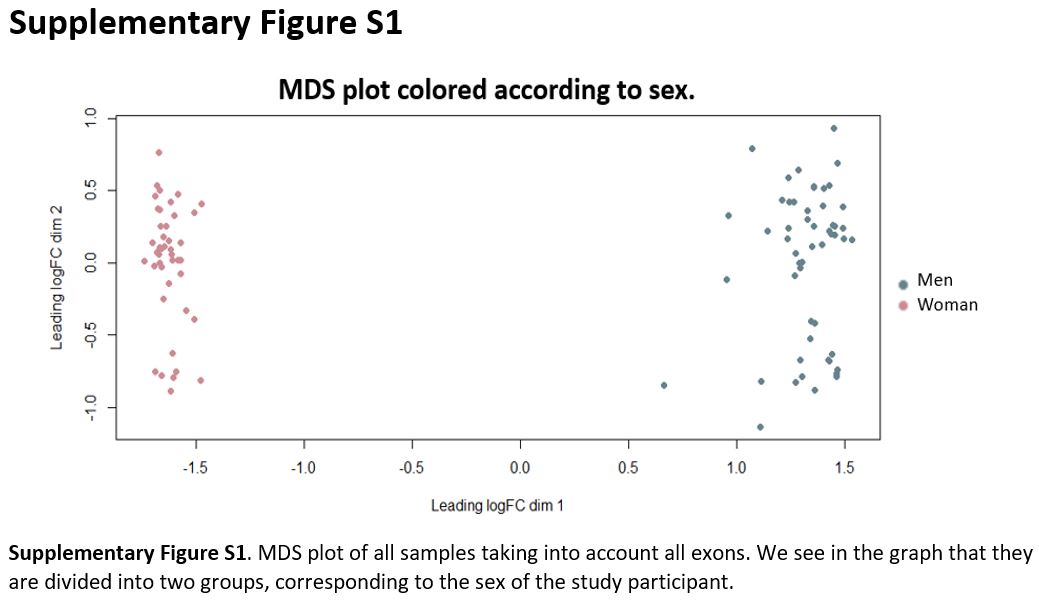

Supplement: Supplementary file 1 — Additional file 1. [file 12885_2022_10459_MOESM1_ESM.jpg]

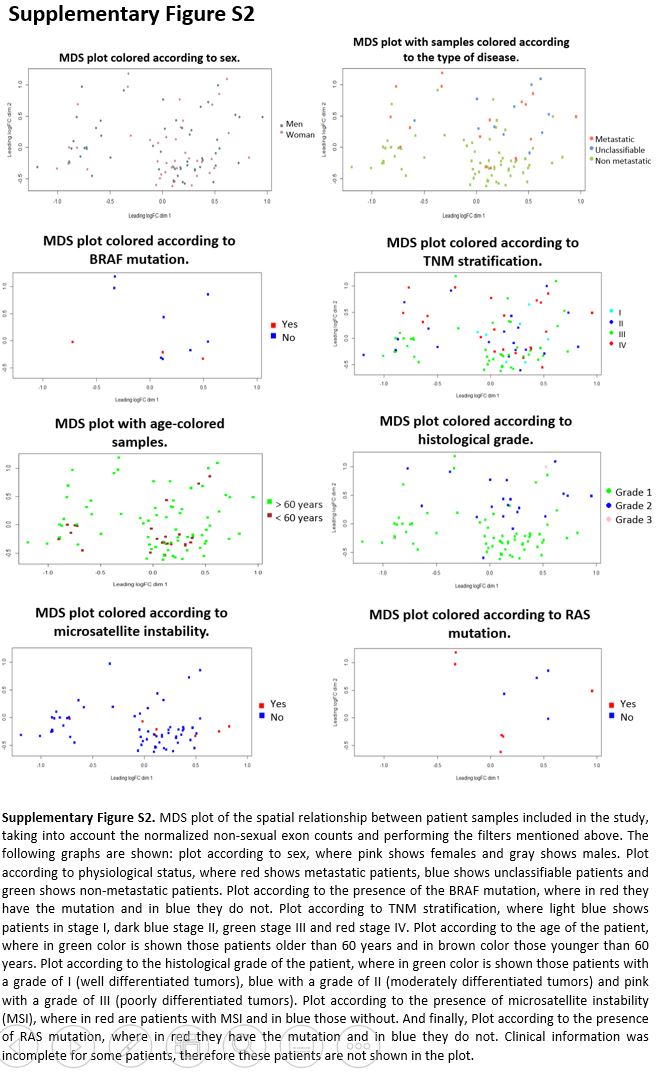

Supplement: Supplementary file 2 — Additional file 2. [file 12885_2022_10459_MOESM2_ESM.jpg]

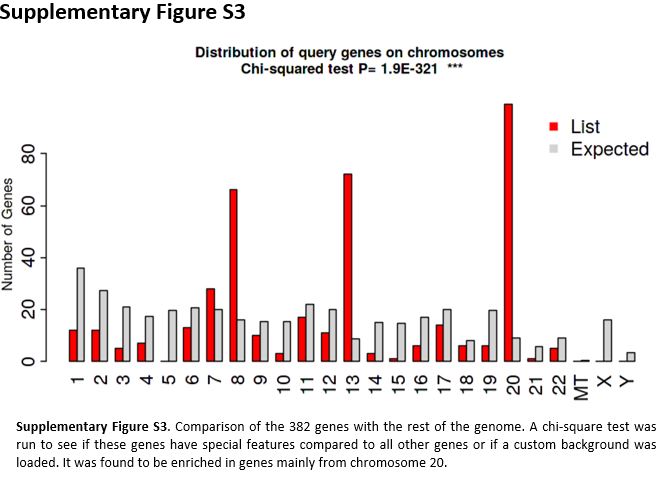

Supplement: Supplementary file 3 — Additional file 3. [file 12885_2022_10459_MOESM3_ESM.jpg]

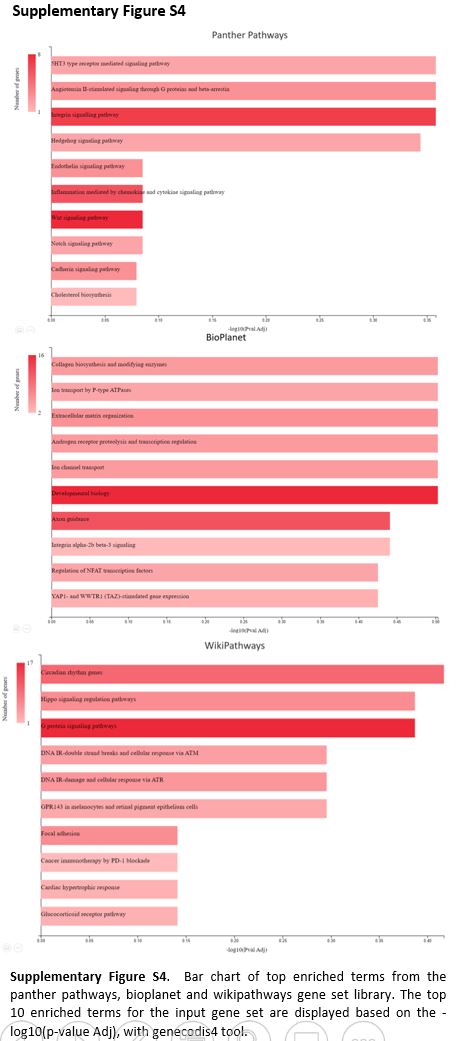

Supplement: Supplementary file 4 — Additional file 4. [file 12885_2022_10459_MOESM4_ESM.jpg]
