# Supplementary material for: Differential presence of exons (DPE): sequencing liquid biopsy by NGS. A new method for clustering colorectal Cancer patients
Source: BMC Cancer. 2023 Jan 3;23:2. doi: 10.1186/s12885-022-10459-w (PMC9808981; doi:10.1186/s12885-022-10459-w)
Supplement: Supplementary file 5 — Additional file 5. [file 12885_2022_10459_MOESM5_ESM.docx]

**SUPPLEMENTARY TABLES**

**Supplementary Table S1.** Descriptive analysis of age according to physiological state.

| TYPE | Mean (years) | standard deviation | variance | median |
| --- | --- | --- | --- | --- |
| Metastatic | 69 | 18 | 311 | 71 |
| Non-metastatic | 69 | 12 | 138 | 68 |
| Unclassifiable | 81 | 9 | 75 | 81 |

**Supplementary Table S2.** Probability of unclassifiable patients belonging to the study groups.

|  | Metastatic | Non-metastatic | Healthy |
| --- | --- | --- | --- |
| PBL04 | 0.86 | 0.11 | 0.03 |
| PBL08 | 0.14 | 0.70 | 0.16 |
| PBL12 | 0.13 | 0.74 | 0.13 |
| PBL15 | 0.06 | 0.77 | 0.17 |
| PBL23 | 0.40 | 0.53 | 0.07 |
| PBL28 | 0.22 | 0.72 | 0.06 |
| PBL57 | 0.25 | 0.62 | 0.13 |
| PBL58 | 0.22 | 0.59 | 0.19 |
| PBL63 | 0.36 | 0.52 | 0.12 |
| PBL66 | 0.65 | 0.30 | 0.05 |
| FJD087 | 0.02 | 0.90 | 0.08 |

**Supplementary Table S3.** Sensitivity and specificity of the model for the different groups.

|  | Metastatic | Non metastatic | Healthy |
| --- | --- | --- | --- |
| Sensitivity | 0.20 | 0.83 | 0.87 |
| Specificity | 1 | 0.70 | 0.88 |

**Supplementary Table S4.** Enrichment analysis based on hypergeometric distribution followed by FDR correction with ShinyGO tool.

| *Enrichment FDR* | *Genes in list* | *Total genes* | *Functional Category* |
| --- | --- | --- | --- |
| 2.4E-32 | 47 | 231 | Chr20q11 |
| 1.1E-15 | 40 | 388 | Chr20q13 |
| 8.3E-13 | 99 | 2455 | Kidney cancer |
| 8.0E-12 | 22 | 132 | NIKOLSKY BREAST CANCER 8Q12 Q22 AMPLICON |
| 8.0E-12 | 23 | 149 | NIKOLSKY BREAST CANCER 20Q12 Q13 AMPLICON |
| 4.6E-09 | 38 | 568 | Liver cancer |
| 6.2E-09 | 32 | 413 | Breast cancer |
| 3.8E-07 | 10 | 31 | NIKOLSKY BREAST CANCER 20Q11 AMPLICON |
| 1.1E-06 | 18 | 157 | NIKOLSKY BREAST CANCER 8Q23 Q24 AMPLICON |
| 1.9E-05 | 22 | 284 | Endometrial cancer |
| 2.0E-05 | 235 | 10658 | Carcinoma |
| 8.6E-05 | 28 | 487 | Melanoma |
| 1.1E-04 | 41 | 939 | PAX3-FKHR 20663909 ChIP-Seq RHABDOMYOSARCOMA Human |
| 2.2E-04 | 12 | 93 | NIKOLSKY MUTATED AND AMPLIFIED IN BREAST CANCER |
| 2.7E-04 | 50 | 1325 | ZNF217 24962896 ChIP-Seq MCF-7 Human |
| 4.1E-04 | 15 | 163 | Chr8q21 |
| 1.6E-03 | 24 | 439 | Skin cancer |
| 5.2E-03 | 53 | 1601 | AR 25329375 ChIP-Seq VCAP Human |
| 8.1E-03 | 55 | 1715 | CSB 26484114 Chip-Seq FIBROBLAST Human |
| 1.1E-02 | 27 | 600 | AHR 22903824 ChIP-Seq MCF-7 Human |
| 1.1E-02 | 11 | 115 | Large intestine cancer |
| 1.2E-02 | 12 | 140 | Chr8q22 |
| 1.4E-02 | 10 | 97 | Antimicrobial peptides |
| 1.4E-02 | 65 | 2216 | SMAD4 21799915 ChIP-Seq A2780 Human |
| 1.6E-02 | 7 | 43 | Chr8q23 |
| 1.8E-02 | 20 | 381 | Lung cancer |
| 1.8E-02 | 15 | 229 | Chr13q12 |
| 2.4E-02 | 22 | 458 | MethyCancer Lung Non-small cell carcinoma |
| 2.6E-02 | 11 | 130 | Pancreatic cancer |
| 3.5E-02 | 49 | 1564 | Hsa-miR-4251 target gene |
